# Harmonization of nomenclature for molecular imaging metrics of tumour burden: molecular tumour volume (MTV), total lesion activity (TLA) and total lesion fraction (TLF)

**DOI:** 10.1007/s00259-021-05613-8

**Published:** 2021-11-13

**Authors:** Jean-Mathieu Beauregard, Arman Rahmim

**Affiliations:** 1 Department of Radiology and Nuclear Medicine, and Cancer Research Centre, Université Laval, Quebec City, Canada; 2 Department of Medical Imaging, CHU de Québec–Université Laval, Quebec City, Canada; 3 Oncology Axis, CHU de Québec–Université Laval Research Centre, Quebec City, Canada; 4 Departments of Radiology and Physics, University of British Columbia, Vancouver, Canada; 5 Department of Integrative Oncology, BC Cancer Research Institute, Vancouver, Canada

Dear Sir,

We read with interest the Letter to the Editor “It is time for the nuclear medicine community to define a unit for the total lesion glycolysis (TLG) and similar metrics” by Duarte and Sapienza [1]. We have had a related reflection in the context of the ongoing accelerated developments in the fields of molecular imaging and theranostics. Volumetric metrics of tumour burden (e.g. metabolic tumour volume, MTV, as well as TLG and its multiple name variants) are indeed increasingly used imaging biomarkers and found to be even more useful than those of uptake intensity (e.g. standardized uptake value, SUV) in a number of applications, including in theranostics [2, 3].

^18^F-fluorodeoxyglucose (FDG)-PET has had a dominating presence for decades. Hence, references to metabolism and glucose consumption have claimed and narrowed down the concepts behind MTV and TLG [4]. In fact, these metrics are increasingly used in imaging of non-metabolic radiopharmaceuticals, including theranostic ones, not only with PET but also with SPECT [3, 5]. Regarding MTV, we find that a simple renaming such as *molecular tumour volume* would appropriately define the tumour volume defined by a non-metabolic ligand. An added benefit is that it leaves the familiar MTV acronym intact. Conversely, TLG needs a rebranding. To define the product of SUV_mean_ and MTV, we suggest *total lesion activity* (TLA), which is a more neutral term, as *activity* does not refer to the specific type of target involved.

As for the units of each metric, that of SUV needs to be sorted out first. There is an ongoing disagreement in the community as to whether SUV has for unit g/mL or is unitless under the assumption that the average body density is close to 1 g/mL, i.e. the density of water. Even the two authors of this letter are divided on this question, one preferring the former (AR) and the other the latter scheme (JMB)! To solve this issue, we propose to explicitly insert the constant reflecting the assumption (i.e. 1 g/mL) in the denominator of the SUV formula, thus cancelling the units of SUV. Accordingly, not only MTV, but also TLG and TLA (i.e. the product SUV_mean_ and MTV) could all be expressed in a unit of volume. In fact, TLG and TLA can be seen as a normalized tumour volume (i.e. the tumour volume if its SUV was normalized to 1). Although a trivial issue, if we had to choose one such unit, we would favour mL over cm^3^ or cc, since Bq/mL is the common activity concentration unit in PET DICOM headers. As a side note, online search returns significantly more results when searching “metabolic tumor volume” or “total lesion glycolysis” with “mL”, rather than with “cm^3^” or “cc”, suggesting a general preference for the former.

More importantly, we wish to propose a practical, alternate metric for molecular tumour burden: *total lesion fraction* (TLF). TLF is the total integrated activity, say in Bq, in the lesion (whether a single lesion or a collection, e.g. whole-body tumour burden) divided by decay-corrected administered activity (in same units as above). In current viewing software, this can easily be obtained by dividing TLG or TLA by the estimated body volume in mL (based on either body weight or lean body weight as used to compute SUV and TLG or TLA). TLF is a unitless metric with a predefined and intuitive scale, that is, from 0 to 100%. By contrast, the TLG or TLA scale is different for each patient, as it theoretically spans up to the estimated patient volume.

We propose to routinely report TLF in addition to other metrics such as MTV and TLA. A feature of TLF is that it is independent of body habitus indices. TLF may in fact better illustrate the relative importance of the tumour burden for patients of different sizes and thus facilitate interpatient comparisons. For example, two patients having an identical TLA of 10,000 mL would be deemed to have the same tumour burden by this metric. However, if these patients weigh 50 kg and 100 kg, they will have TLFs of 20% and 10%, respectively. In such a scenario, TLF may better convey that the former patient has a larger relative tumour burden and may thus have potentially poorer outcome given relatively smaller reserve by the body to encounter the tumour load, which can be investigated in different outcome prediction studies. In other words, it is plausible that TLF may improve prediction of outcome relative to TLA (and other metrics such as SUV), and by more routinely reporting this metric, such a hypothesis can be further evaluated for different diseases and radiopharmaceuticals. Furthermore, when used to assess whole-body tumour burden, TLF becomes a straightforward measure of the tumour sink effect, that is, the fraction of administered activity sequestered in the tumour that is not available for uptake by healthy tissues [6]. This has implication for radiopharmaceutical therapy, as a large burden of radiopharmaceutical-avid tumour is associated with decreased healthy tissues absorbed doses per unit administered activity, leaving room for escalation of administered activity (and thus of tumour absorbed dose) in a personalized approach [7].

Metabolic response using FDG-PET is commonly based on change in maximum or peak SUV, as in PERCIST [8]. However, SUV may not be as appropriate to assess molecular response using theranostic radiopharmaceuticals, and variations in MTV, TLA and potentially TLF appear more promising for this purpose [2, 3]. In line with this, we recently proposed the Theranostic Response Criteria In Solid Tumours (THERCIST) that is applicable to both PET and quantitative SPECT [9]. Interestingly, we have found that the early TLF-based response assessed with ^177^Lu-DOTATATE post-treatment quantitative SPECT accurately predicted the late MTV-based response assessed with ^68^Ga-DOTATATE PET [10].

In conclusion, to widen the scope of MTV and TLG beyond FDG-PET, we propose replacing *metabolic* with *molecular* within MTV and renaming TLG to TLA (and to express all these in mL). Furthermore, we propose TLF as a simple, unitless and intuitive way to further quantify overall tumour avidity especially in the era of theranostics.

Jean-Mathieu Beauregard.

Arman Rahmim.
